# Supplementary material for: Deletion of wfs1 Impairs Oligodendrocyte Precursor Cells Dorsal Distribution and Myelination Through the wfs1-hmgcs1 Axis in Zebrafish
Source: Biology (Basel). 2026 Aug 16;15(16):1402. doi: 10.3390/biology15161402 (PMC13510151; doi:10.3390/biology15161402)
Supplement: Supplementary file 1 [file biology-15-01402-s001.zip › File S1-Transcriptome sequencing data and differentially expressed genes (DEGs) in wfs1-- zebrafish at 5 dpf/2.KEGG/wfs1KOvsWT/UP/src/dre04260.html]

dre04260


- dre:100535196

- Up regulated genes

100535196(2.10467205781268)

- dre:677750

- Up regulated genes

677750(1.37913343503877)

- dre:677750

- Up regulated genes

677750(1.37913343503877)

- dre:556638

- Up regulated genes

556638\_2(6.35906141157549)

- dre:447942

- Up regulated genes

447942(1.89918802415248)

- dre:794522

- Up regulated genes

794522(1.07749660517023)
- dre:114457

- Up regulated genes

114457\_1(1.40796876982558)

Close
